# Broiler Responses to Dietary Fibre Sources at Different Ages: Effects on Growth Performance, Nutrient Digestibility, Blood Parameters and Intestinal Morphology

**DOI:** 10.1002/vms3.70471

**Published:** 2025-06-19

**Authors:** Ali Asghar Kardel, Mohammad Kazemifard, Mansour Rezaei, Asadollah Teimouri Yansari

**Affiliations:** ^1^ Department of Animal Science Sari Agricultural Sciences and Natural Resources University Sari Iran

**Keywords:** broiler, dietary fibre, different ages, morphology, performance

## Abstract

**Background:**

Previous studies investigated the effect of different levels of fibre sources, but little research is available on the effect of a bird's age on the type of fibre source.

**Objectives:**

The study aimed to determine the effect of rice hulls (RHs) and sugar beet pulp (SBP) on growth performance, nutrient digestibility, blood parameters and intestinal morphology of broilers at different ages.

**Methods:**

A total of 160 newly hatched Ross 308 male broiler chicks were randomly assigned to 4 dietary groups, each with 4 replicates and 10 chicks per replicate. Dietary treatments included: T1: control diet without any additions at grower and finisher periods; T2: diet containing 3% RH in the grower period and 3% SBP in the finisher period; T3: diet containing 3% SBP in the grower period and 3% RH in the finisher period; and T4: diet containing 1.5% RH and 1.5% SBP in the grower and finisher periods. Average growth performance during this period and nutrient digestibility, blood parameters and ilium morphology were evaluated at Days 24 and 42.

**Results:**

Feed conversion ratio (FCR) of broilers significantly improved in the T2 group from 11 to 24 days and body weight gain (BWG) in T2 and T4 from 11 to 42 days of age (*p* < 0.05). At 42 days of age, the greatest dry matter (DM), organic matter (OM) and neutral detergent fibre (NDF) digestibility were observed in the T4 group in comparison to the T3 groups (*p* < 0.05). The ilium villus height–to–crypt depth (VH/CD) ratio increased at 24 days of age in the T1 and T2 groups (*p* < 0.05).

**Conclusions:**

Our study showed that the T2 and T4 groups might enhance broiler growth performance through increasing nutrient digestibility and ilium morphology during Days 11 to 42 days of age.

## Graphical abstract

Inclusion of rice hulls during the grower period and sugar beet pulp during the finisher period improved FCR and BWG in broiler chickens. Combined fibre sources enhanced nutrient digestibility and increased villus height–to–crypt depth ratio.

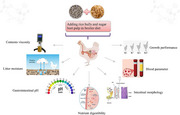

## 1 Introduction

Carbohydrates are the most abundant component of poultry diets and one of the least noticeable nutrients in broiler diets, particularly dietary fibre (DF). Commercial diets usually include 20–30 g of crude fibre (CF) per kilogram, which is necessary for optimal gastrointestinal organ activity (Hetland et al. 2004; Jiménez‐Moreno, González‐Alvarado, de Coca‐Sinova, et al. 2009; Choct 2015). A moderate level of DF may enhance growth performance by promoting digestive system development, improving nutrient digestibility and stimulating digestive enzyme activity (Mateos et al. 2012; Tejeda and Kim 2021).

Physicochemical characteristics of fibrous compounds, including solubility, viscosity, water‐holding capacity, fermentability and bulkiness, may have varying impacts on nutrient digestibility and gut development in non‐ruminant animals (Montagne et al. 2003). DF is divided into soluble fibre and insoluble fibre based on its solubility in water. For example, rice hulls (RHs) and oat hulls are composed of insoluble fibre (87% and 83%), and sugar beet pulp (SBP) and pectin are composed of soluble fibre (28% and 65%), respectively (Tejeda and Kim 2021). The inclusion of 3% sunflower hulls and oat hulls in broiler diets has been reported to improve growth performance, specifically feed conversion ratio (FCR) and average daily gain (Jiménez‐Moreno et al. 2016). Sadeghi et al. (2015) observed that broilers consuming 1.5% SBP and 1.5% RH had lower daily feed intake (FI) than those fed a diet containing 3% SBP from 14 to 28 days of age. Supplementation of broiler diets with 8% CF using soya bean hulls as the fibre source increased villus height (VH) in the duodenum during the first 20 days of age (Tejeda and Kim 2020). According to Sadeghi et al. (2015), broilers given the 3% SBP diet had lower VH in the duodenum and ileum than in the control group. Furthermore, Hetland and Svihus (2001) observed that broiler ileal digestion of starch improved by including cellulose and oat hulls in the diet. Moreover, Jiménez‐Moreno, González‐Alvarado, González‐Serrano et al. (2009) found that total tract apparent digestibility of lipids in young chicks improved by including SBP or oat hulls. On the other hand, a reduced feed passage rate in older birds and exposure to microbial fermentation in the caeca for a longer time could improve the digestibility of DF (Shires et al. 1987). Mateos et al. (2012) found that increased retention time of ingested feed in the gizzard increases its activity, potentially improving digestion and nutrient absorption. The 15‐day‐old broiler chicks exhibited lower digestibility of total non‐starch polysaccharides and oligosaccharides compared to adult cockerels, which was attributed to their higher intestinal absorption efficiency (Carré et al. 1995). Therefore, fibre digestibility may vary depending on the bird age. Sadeghi et al. (2015) studied the influence of RHs and SBP on choice in broiler chickens aged 1–42, but less‐considered fibre types such as RHs and SBP at different ages require further research.

There are few published works on the effects of DF sources on the performance of broiler chickens at different ages. This study aimed to evaluate the effects of two different fibre sources (RHs and SBP) on ileal morphology, blood parameters, growth performance and nutrient digestibility in broilers at different ages.

## 2 Materials and Methods

### 2.1 Birds, Housing and Treatments

The research involved 160 (*n* = 160) 1‐day‐old male Ross 308 broiler chicks. The chicks had been distributed in 4 treatments with 4 replicates (10 chicks/replicate; *n* = 40 per group). Among the experimental treatments were: T1: control diet without any additions at grower and finisher periods; T2: diet containing 3% RH in the grower period and 3% SBP in the finisher period; T3: diet containing 3% SBP in the grower period and 3% RH in the finisher period; T4: diet containing 1.5% RH and 1.5% SBP in the grower and finisher periods. The fibre sources were analysed for chemical composition (Table 1), and their particle size was determined using the standard method ASAE (1995). The experimental diets were formulated for three periods: starter, grower and finisher from 1 to 10, 11 to 24 and 25 to 42 days of age, respectively. The ingredients and chemical composition of the experimental diets are shown in Table 2. Throughout the trial, feed and water were given ad libitum to the chicks. The room temperature was set to 32°C and gradually decreased by 3°C per week until it reached 22°C.

**TABLE 1:** Chemical composition (dry matter basis, %), physical and buffering properties of the fibre sources.

Items	Sugar beet pulp	Rice hull
**Chemical composition** ^a^		
DM	85.40	91.80
CP	10.70	3.70
EE	4.59	3.60
Ash	8.80	23.20
NDF	49.10	67.80
ADF	20.10	51.70
NFC	26.80	1.70
**Physical characteristics** ^b^		
Screen size (µm)		
>4760	1.20	—
3360	5.40	—
2380	19.20	—
2000	11.40	1.70
1680	15.40	10.80
1190	22.10	25.90
841	12.00	20.30
500	8.15	14.80
<500	5.15	26.50
GMD (µm)	1577	777
GSD	1.89	2.11
WHC (mL/g of DM)	4.81	2.85
**Buffering characteristics** ^c^		
Initial pH	5.15	5.98
Base‐buffering capacity	13.40	10.70
Acid‐buffering capacity	60.60	19.10

^a^ Chemical composition: DM, dry matter; CP, crude protein; EE, ether extract; NDF, neutral detergent fibre; ADF, acid detergent fibre; NFC, non‐fibre carbohydrate = 100 − (CP + Ash + EE + NDF).

^b^ Physical characteristics: GDM, geometric diameter mean; GSD, geometric standard deviation; WHC, water‐holding capacity.

^c^ Buffering characteristics: initial pH, determined using 0.5 g of DM suspended in 50 mL of distilled water; base‐buffering capacity, calculated as the amount of micro‐equivalents of NaOH required to increase the pH of 0.5 g of DM sample suspended in 50 mL of distilled water from the initial pH to pH 7 divided by pH change; and acid‐buffering capacity, calculated as the amount of micro‐equivalents of HCl required to reduce the pH of 0.5 g of DM sample suspended in 50 mL of distilled water from pH 7 to pH 4 divided by pH change.

**TABLE 2:** Ingredients and chemical composition of the experimental diet at different ages (dry matter basis, %).

Items	Starter (1–10)	Grower (11–24 days old)				Finisher (25–42 days old)			
		CON^a^	RH^b^	BP^c^	RH/BP^d^	CON	RH	BP	RH/BP
**Ingredients (%)**									
Corn grain	51.02	54.42	52.16	52.71	52.43	59.21	56.94	57.50	57.22
Soya bean meal (44% CP)	40.71	35.35	36.08	35.54	35.81	30.24	30.97	30.44	30.71
Sand	—	3.00	—	—	—	3.00	—	—	—
Rice hull	—	—	3.00	—	1.50	—	3.00	—	1.50
Sugar beet pulp	—	—	—	3.00	1.50	—	—	3.00	1.50
Soya bean oil	3.61	2.95	4.56	4.46	4.51	3.58	5.18	5.08	5.13
Dicalcium phosphate	1.76	1.57	1.58	1.59	1.58	1.41	1.42	1.43	1.42
CaCo_3_	1.20	1.11	1.03	1.10	1.07	1.02	0.95	1.01	0.98
Salt	0.34	0.34	0.34	0.34	0.34	0.34	0.35	0.35	0.35
Mineral premix^e^	0.25	0.25	0.25	0.25	0.25	0.25	0.25	0.25	0.25
Vitamin premix^f^	0.25	0.25	0.25	0.25	0.25	0.25	0.25	0.25	0.25
l‐Lysine. HCl	0.34	0.31	0.29	0.30	0.30	0.30	0.29	0.29	0.29
l‐Threonine	0.14	0.12	0.12	0.12	0.12	0.10	0.09	0.09	0.09
dl‐Methionine	0.38	0.33	0.34	0.34	0.34	0.30	0.31	0.31	0.31
**Calculate nutrient analysis**									
Metabolism energy (kcal/kg)	2910	2945	2945	2945	2945	3040	3040	3040	3040
Crude protein (%)	22.30	20.40	20.40	20.40	20.40	18.50	18.50	18.50	18.50
ME/CP	130	144	144	144	144	164	164	164	164
Acid linoleic (%)	3.16	2.94	3.66	3.62	3.64	3.35	4.07	4.03	4.05
Crude fibre (%)	3.97	3.73	4.99	4.25	4.62	3.48	4.74	4.00	4.37
Ether extract	5.87	6.11	6.83	6.77	6.80	6.98	7.42	7.33	7.38
Calcium (%)	0.96	0.87	0.87	0.87	0.87	0.79	0.79	0.79	0.79
Available phosphorous (%)	0.48	0.43	0.43	0.43	0.43	0.39	0.39	0.39	0.39
Sodium (%)	0.16	0.16	0.16	0.16	0.16	0.16	0.16	0.16	0.16
Chloride (%)	0.31	0.30	0.30	0.30	0.30	0.30	0.30	0.30	0.30
Potassium (%)	0.96	0.88	0.88	0.88	0.88	0.79	0.79	0.79	0.79
DCAB (meq/kg)^g^	247	224	224	221	223	201	201	199	200
Methionine (%)	0.68	0.62	0.62	0.62	0.62	0.56	0.56	0.56	0.56
Lysine (%)	1.44	1.29	1.29	1.29	1.29	1.16	1.16	1.16	1.16
Cystine (%)	0.35	0.32	0.32	0.32	0.32	0.30	0.30	0.30	0.30
Methionine + cystine (%)	1.08	0.99	0.99	0.99	0.99	0.91	0.91	0.91	0.91
Threonine (%)	0.97	0.88	0.88	0.88	0.88	0.78	0.78	0.78	0.78
Arginine (%)	1.47	1.32	1.32	1.32	1.32	1.18	1.19	1.18	1.18
Tryptophan (%)	0.27	0.24	0.24	0.24	0.24	0.21	0.21	0.21	0.21

^a^ CON, control diet (basal diet).

^b^ RH, basal diet supplemented with rice hulls.

^c^ BP, basal diet supplemented with sugar beet pulp.

^d^ RH/BP, basal diet supplemented with a combination of rice hulls and sugar beet pulp.

^e^ Mineral premix per kg of diet: Mn (MnSO_4_·H_2_O, 32.49% Mn), 75 mg; I (KI, 58% I), 1 mg; Cu (CuSO_4_·5H_2_O), 8 mg; Fe (FeSO_4_·7H_2_O, 20.09% Fe), 50 mg; Se (NaSeO_3_, 45.56% Se), 0.2 mg.

^f^ Vitamin premix per kg of diet: vitamin A (retinol).8000 IU; vitamin D3 (cholecalciferol), 2000 IU; vitamin E (tocopheryl acetate), 12.5 IU; vitamin K3, 2 mg; vitamin B1, 1.8 mg; vitamin B2, 6 mg; B5, 10 mg; B6, 2.8 mg; niacin, 40 mg; biotin, 0.1 mg; folic acid, 1 mg.

^g^Dietary cation–anion balance (Na + K − Cl).

### 2.2 Growth Performance

Body weight gain (BWG) and FI have been determined at 11–24, 25–42 and 11–42 days of age. The FCR in each phase was determined by dividing the intake of feed by BWG.

### 2.3 Blood Parameters

Samples of blood were taken from the wing veins of two birds per replicate and transferred to EDTA‐sterile tubes to assay glucose (GLU), triglyceride (TG) and total‐cholesterol (T‐CHO) concentrations at 42 days of the experiment. The samples have been centrifuged at 3000*g* for 10 min at 4°C, according to Friedewald et al. (1972). Moreover, very low‐density lipoprotein (VLDL) was estimated by dividing TG by five (Musa et al. 2007).

### 2.4 Measuring Gastrointestinal pH and Litter Moisture

The moisture content of the litter was measured during the grower and finisher periods (AOAC 2000). Gizzard and ilium digesta from two birds per pen were separated, pooled and homogenized in 50‐mL vessels to measure the pH by a portable pH meter (González‐Alvarado et al. 2008).

### 2.5 Measurement of Total Tract Nutrient Digestibility

The total tract nutrient digestibility was measured with chromium oxide (3 g of Cr_2_O_3_/kg of diet) as an indigestible marker in the experimental diets at Days 24 and 42. The diets contained chromium oxide for 72 h at adaptation, and after separate excreted samples were collected for 48 h. The AOAC (1995) processes were utilized for determining the chemical composition of the excreta and diet samples, including dry matter (DM), ether extract (EE), crude protein (CP), organic matter (OM) and neutral detergent fibre (NDF). The marker in the feed and excreta samples was calculated by spectrophotometer (Fenton and Fenton 1979). The nutritional digestibility was calculated using the following formula:

$$D\%=100\;-\;\left[100\;\times \;\left(A/B\right)\;\times \;\left(C/E\right)\right]$$

where *D* is the digestibility, *A* is the chromium oxide in feed (%), *B* is the chromium oxide in excreta (%), *C* is the nutrient concentration in excreta (%) and *E* is the nutrient concentration in feed (%).

### 2.6 Ileal Morphometric Measurement

At Days 24 and 42, two birds per pen were weighed and slaughtered. Following evisceration, the ileum of the small intestine was separated, washed with physiological serum and fixed with 10% formalin solution. The tissue passage included three stages of dehydration, transpiration and saturation of the samples, which were conducted using alcohol, xylene and melted paraffin, respectively. After fixing, the samples were prepared and stained with haematoxylin and eosin methods. Finally, an electron microscope was used to determine the villus thickness (VH), villus width (VW) and crypt depth (CD) (Bradley et al. 1994). The formula for calculating villus surface area (VSA) is as follows (Sakamoto et al. 2000):


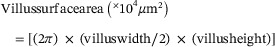




### 2.7 Relative Ilium Length and Ilium Contents Viscosity

The length of ilium had been determined and then estimated using the following formula:

$$\begin{array}{ccc} & & \mathrm{Relative}\mathrm{intestinal}\mathrm{length}\left(\mathrm{cm}/\mathrm{kg}\right)\\ & & \;=\mathrm{ilium}\mathrm{length}\left(\mathrm{cm}\right)/\mathrm{live}\mathrm{body}\mathrm{weight}\left(\mathrm{kg}\right) \end{array}$$

To measure viscosity, the samples were taken from ileal digests at 24 and 42 days of age. The contents (3 g) were transferred to a 15 cc tube and centrifuged at 12,500*g* for 5 min. The supernatant was separated and reached 16 cc volume by adding water. Viscosity was then measured using a Brookfield digital viscometer (LVDVE 230) at 12 rpm with spindle 21 at 25°C after 10 s (Perryman et al. 2013).

### 2.8 Statistical Analysis

The results from this experiment have been evaluated in a completely randomized design using SAS software GLM methods (SAS Institute 2001). The mean differences were compared using the Duncan multiple‐range test. The level of significance was 0.05.

## 3 Results

### 3.1 Growth Performance

Table 3 shows the effect of experimental treatments on the growth performance of broilers. The T1 group had the lowest FI compared to the other groups at 11–24 days (*p* < 0.05). In the finisher and whole period (25–42 and 11–42 days of age), the birds fed T3 had the lowest FI compared to the other groups (*p* < 0.05). From 11 to 24 days, the T1 group had the lowest BWG compared to the T2 and T3 groups (*p* < 0.05). Broilers in the T4 group had significantly higher BWG than the other groups at 25–42 days of age (*p* < 0.05). BWG was greater (*p* < 0.05) in chicks at 11–42 days of age when fed the T2 and T4 diets in comparison to the T1 and T3 groups. From 11 to 24 days, the FCR improved in broilers that received T2 diets compared with other groups (*p* < 0.05). From 25 to 42 days, the T1 group had the highest FCR compared to the T3 and T4 groups (*p* < 0.05). Broilers in the T1 group had a significantly higher FCR than the other groups at 11–42 days of age (*p* < 0.05).

**TABLE 3:** Effect of experimental treatments on the growth performance of broiler at different ages.

	Treatments					
Items	T1	T2	T3	T4	SEM	*p* value
**11–24 days**						
FI (g/bird/day)	1157^c^	1300^b^	1329^ab^	1359^a^	17.646	<0.0001
BWG (g/bird/day)	674^c^	822^a^	776^ab^	733^cb^	24.034	0.0058
FCR (g/g)	1.71^b^	1.58^c^	1.71^b^	1.85^a^	0.036	0.0024
**25–42 days**						
FI (g/bird/day)	2881^a^	2799^a^	2508^b^	2877^a^	33.276	<0.0001
BWG (g/bird/day)	1353^b^	1424^b^	1324^b^	1592^a^	38.744	0.0017
FCR (g/g)	2.13^a^	1.97^ab^	1.90^b^	1.80^b^	0.065	0.0280
**11–42 days**						
FI (g/bird/day)	4039^b^	4100^b^	3849^c^	4236^a^	41.687	0.0002
BWG (g/bird/day)	2028^b^	2247^a^	2100^b^	2325^a^	37.323	0.0004
FCR (g/g)	1.99^a^	1.82^b^	1.83^b^	1.82^b^	0.0365	0.0176

*Note*: Dietary treatments: T1: control diet (basal diet); T2: diet containing 3% rice hull in grower period (11–24 days) and 3% sugar beet pulp in finisher period (25–42 days); T3: diet containing 3% sugar beet pulp in grower period (11–42 days) and 3% rice hull in finisher period (25–42 days); T4: diet containing 1.5% rice hull and 1.5% sugar beet pulp in grower period (11–24 days) and finisher period (25–42 days). Different letters denote significant difference (*p* < 0.05).

Abbreviations: BWG, body weight gain; FCR, feed conversion ratio; FI, feed intake; SEM, standard error of the means.

### 3.2 Blood Parameters

As shown in Table 4, GLU concentration at 24 days and T‐CHO concentration at 42 days of age had significant effects by experimental treatments. At 24 days of age, the T2 group had significantly decreased GLU levels compared to the other groups (*p* < 0.05). A significantly lower T‐CHO concentration was observed in broilers receiving the T3 diet compared to the other groups at 42 days of age (*p* < 0.05). The results revealed no significant differences in TG and VLDL concentrations at 24 and 42 days of age.

**TABLE 4:** Effect of experimental treatments on some blood parameters of broiler at different ages (mg/dL).

	Treatments					
Items	T1	T2	T3	T4	SEM	*p* value
**24 days**						
GLU	225^a^	206^b^	222^a^	225^a^	3.779	0.0355
TG	42.33	41.33	46.00	37.33	2.179	0.1920
T‐CHO	140	143	138	125	6.290	0.3603
VLDL	8.46	8.26	9.20	7.46	0.435	0.1920
**42 days**						
GLU	219	215	217	204	9.647	0.7013
TG	49.75	37.66	38.25	49.33	3.649	0.0856
T‐CHO	144^a^	136^a^	121^b^	135^a^	3.133	0.0062
VLDL	9.95	7.53	7.65	9.86	0.583	0.0856

*Note*: Dietary treatments: T1: control diet (basal diet); T2: diet containing 3% rice hull in grower period (11–24 days) and 3% sugar beet pulp in finisher period (25–42 days); T3: diet containing 3% sugar beet pulp in grower period (11–42 days) and 3% rice hull in finisher period (25–42 days); T4: diet containing 1.5% rice hull and 1.5% sugar beet pulp in grower period (11–24 days) and finisher period (25–42 days). Different letters denote significant difference (*p* < 0.05).

Abbreviations: GLU, glucose; SEM, standard error of the means; T‐CHO, total‐cholesterol; TG, triglyceride; VLDL, very low‐density lipoprotein.

### 3.3 Gastrointestinal pH and Litter Moisture

The influence of experimental treatments on the gastrointestinal pH and litter moisture of broilers is shown in Table 5. At 24 days of age, the pH of the ilium in the T3 group was significantly lower than in the other groups. At 24 days of age, the T1 group had higher gizzard pH and litter moisture levels than the other groups (*p* < 0.05). Furthermore, litter moisture in the T3 group had been significantly lower compared to the T1 and T4 groups at 42 days of age.

**TABLE 5:** Effect of experimental treatments on gastrointestinal pH and litter moisture of broiler at different ages.

	Treatments					
Items	T1	T2	T3	T4	SEM2	*p* value
**24 days**						
pH ilium	6.71^a^	6.43^b^	5.90^c^	6.85^a^	0.0572	<0.0001
pH gizzard	4.142^a^	3.327^b^	2.917^c^	3.060^bc^	0.1038	<0.0001
Litter moisture (%)	58.33^a^	48.86^bc^	45.82^c^	51.02^b^	1.2188	0.0001
**42 days**						
pH ilium	5.28	5.84	6.55	5.88	0.1870	0.0630
pH gizzard	3.877	3.300	3.717	3.262	0.2136	0.1614
Litter moisture (%)	61.18^ab^	59.64^bc^	57.29^c^	62.54^a^	0.7377	0.0042

*Note*: Dietary treatments: T1: control diet (basal diet); T2: diet containing 3% rice hull in grower period (11–24 days) and 3% sugar beet pulp in finisher period (25–42 days); T3: diet containing 3% sugar beet pulp in grower period (11–42 days) and 3% rice hull in finisher period (25–42 days); T4: diet containing 1.5% rice hull and 1.5% sugar beet pulp in grower period (11–24 days) and finisher period (25–42 days). Different letters denote significant difference (*p* < 0.05).

Abbreviation: SEM, standard error of the means.

### 3.4 Total Tract Nutrient Digestibility

The effect of experimental treatments on the total tract nutrient digestibility of broilers is indicated in Table 6. At 24 days of age, there had been no significant differences in DM, CP, EE, OM and NDF. The DM and OM digestibility were greatest in broilers fed with the T4 diet, whereas the T3 group had the lowest DM and OM digestibility at 42 days of age (*p* < 0.05). The birds fed with the T4 diet had greater NDF digestibility than the T1 and T3 groups at 42 days of age (*p* < 0.05).

**TABLE 6:** Effect of experimental treatments on the total tract nutrient digestibility of broiler at different ages.

	Treatments					
Items (%)	T1	T2	T3	T4	SEM	*p* value
**24 days**						
DM	71.93	70.70	71.25	70.68	1.0378	0.8096
CP	66.00	63.02	68.34	71.24	5.4462	0.7864
EE	65.54	63.79	65.57	69.23	5.5419	0.9809
OM	63.19	53.37	61.56	57.95	3.3560	0.3858
NDF	11.50	9.30	13.07	9.97	0.8891	0.1169
**42 days**						
DM	80.11^ab^	79.30^ab^	77.86^b^	81.80^a^	0.8241	0.0352
CP	69.90	73.97	64.31	75.46	3.5240	0.1340
EE	74.57	78.56	73.56	79.29	2.7920	0.4766
OM	76.08^ab^	75.29^ab^	64.31^b^	80.14^a^	3.5205	0.0466
NDF	11.86^b^	14.16^ab^	11.76^b^	16.17^a^	0.7877	0.0260

*Note*: Dietary treatments: T1:control diet (basal diet); T2: diet containing 3% rice hull in grower period (11–24 days) and 3% sugar beet pulp in finisher period (25–42 days); T3: diet containing 3% sugar beet pulp in grower period (11–42 days) and 3% rice hull in finisher period (25–42 days); T4: diet containing 1.5% rice hull and 1.5% sugar beet pulp in grower period (11–24 days) and finisher period (25–42 days). Different letters denote significant difference (*p* < 0.05).

Abbreviations: CP, crude protein; DM, dry matter; EE, ether extract; NDF, neutral detergent fibre; OM, organic matter; SEM, standard error of the means.

### 3.5 Intestinal Morphology

**FIGURE 1:**
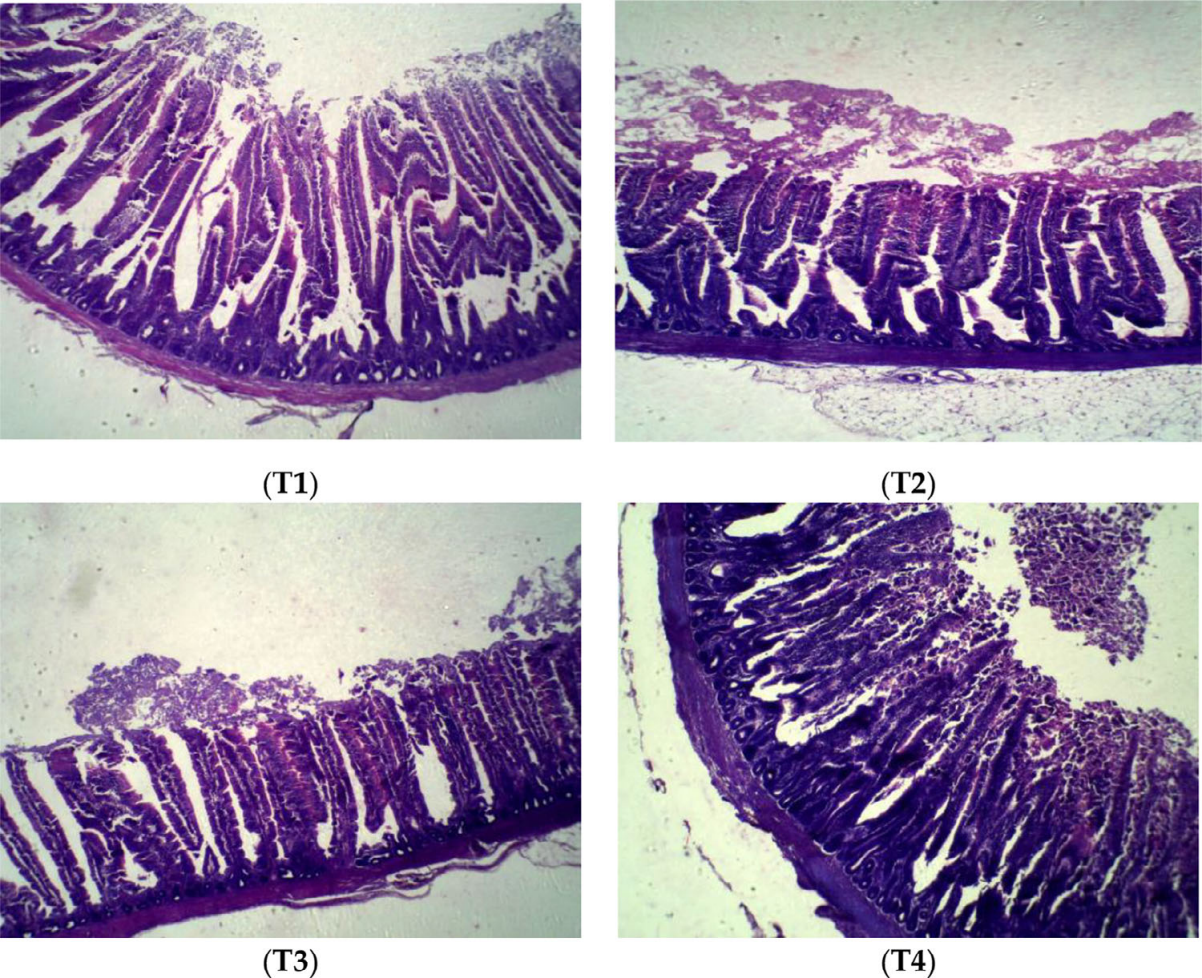
Light microscopy showing the ilium epithelia of broiler at 42 days of age. T1, control diet (basal diet); T2, diet containing 3% rice hull in grower period (11–24 days) and 3% sugar beet pulp in finisher period (25–42 days); T3, diet containing 3% sugar beet pulp in grower period (11–42 days) and 3% rice hull in finisher period (25–42 days); T4, diet containing 1.5% rice hull and 1.5% sugar beet pulp in grower period (11–24 days) and finisher period (25–42 days).

The effect of experimental treatments on the ilium morphology of broilers is shown in Table 7 and Figure 1. The VH of the ilium had been significantly higher in the T2 group at 24 days of age compared to the other groups. The VW and CD in the T1 group were lower (*p* < 0.05) compared to the other groups at 24 days of age. At this age, the VH/CD in ilium was greater in broilers fed with T1 and T2 diets than in the other groups, whereas the T4 group had the lowest VH/CD (*p* < 0.05). The VSA in both the T2 and T3 groups had been significantly higher compared to that in the other groups at 24 days of age. At 42 days of age, the VH, CD and VSA in the ilium were significantly increased in the T1 group compared to the other groups. In the T3 and T4 groups, the VW of the ilium was highest compared to the other groups at 42 days of age (*p* < 0.05). At this age, a significantly higher VH/CD ratio was observed in the T4 group compared to the other treatment groups.

**TABLE 7:** Effect of experimental treatments on the ilium morphology of broiler at different ages.

	Treatments					
Items	T1	T2	T3	T4	SEM	*p* value
**24 days**						
VH (µm)	1161^b^	1340^a^	981^c^	1001^c^	7.186	<0.0001
VW (µm)	105^c^	151^b^	203^a^	147^b^	3.224	<0.0001
CD (µm)	215^d^	260^b^	242^c^	278^a^	2.954	<0.0001
VH/CD	5.45^a^	5.18^a^	4.07^b^	3.61^c^	0.063	<0.0001
VSA (^×^10^4^ µm2)	0.38^c^	0.63^a^	0.62^a^	0.46^b^	0.011	<0.0001
**42 days**						
VH (µm)	1206^a^	1030^c^	880^d^	1108^b^	11.334	<0.0001
VW (µm)	133^b^	157^a^	144^ab^	108^c^	2.760	<0.0001
CD (µm)	284^a^	230^b^	211^b^	225^b^	3.323	<0.0001
VH/CD	4.28^b^	4.50^b^	4.17^b^	5.00^a^	0.078	0.0019
VSA (^×^10^4^ µm2)	0.50^a^	0.50^a^	0.40^b^	0.37^b^	0.010	<0.0001

*Note*: Dietary treatments: T1: control diet (basal diet); T2: diet containing 3% rice hull in grower period (11–24 days) and 3% sugar beet pulp in finisher period (25–42 days); T3; diet containing 3% sugar beet pulp in grower period (11–42 days) and 3% rice hull in finisher period (25–42 days); T4: diet containing 1.5% rice hull and 1.5% sugar beet pulp in grower period (11–24 days) and finisher period (25–42 days). Different letters denote significant difference (*p* < 0.05).

Abbreviations: CD, crypt depth; SEM, standard error of the means; VH, villus height; VH/CD, villus height/crypt depth; VSA, villus surface area; VW, villus width.

### 3.6 Relative Ilium Length and Ilium Contents Viscosity

**FIGURE 2:**
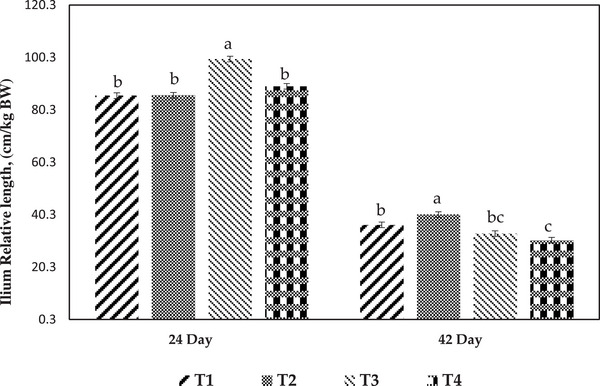
Effect of experimental treatments on the ilium relative length of broiler at different ages. Values are means ± SEM, with four replicates. Different letters denote significant difference (*p* < 0.05). T1, control diet (basal diet); T2, diet containing 3% rice hull in grower period (11–24 days) and 3% sugar beet pulp in finisher period (25–42 days); T3, diet containing 3% sugar beet pulp in grower period (11–42 days) and 3% rice hull in finisher period (25–42 days); T4, diet containing 1.5% rice hull and 1.5% sugar beet pulp in grower period (11–24 days) and finisher period (25–42 days).

**FIGURE 3:**
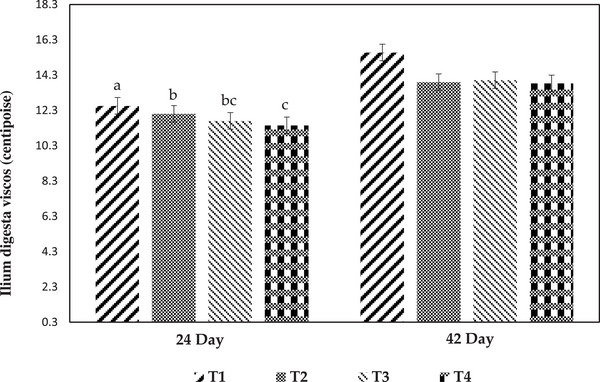
Effect of experimental treatments on the ilium digesta viscosity at different ages. Values are means ± SEM, with four replicates. Different letters denote significant difference (*p* < 0.05). T1, control diet (basal diet); T2, diet containing 3% rice hull in grower period (11–24 days) and 3% sugar beet pulp in finisher period (25–42 days); T3, diet containing 3% sugar beet pulp in grower period (11–42 days) and 3% rice hull in finisher period (25–42 days); T4, diet containing 1.5% rice hull and 1.5% sugar beet pulp in grower period (11–24 days) and finisher period (25–42 days).

Figures 2 and 3 show the relative ilium length and viscosity of the ilium contents during different breeding periods. At 24 days of age, the relative length of the ilium was significantly increased in the T3 group in comparison to the other groups. The greatest relative length of the ilium was observed in broilers fed the T2 diet at the age of 42 days (*p* < 0.05). At this age, there was no significant difference among treatments for the viscosity of ilium contents. The viscosity of the ilium contents significantly decreased in the T4 group compared to the T1 and T2 groups at 24 days of age.

## 4 Discussion

Broiler performance was influenced by DF, although the effects varied depending on the fibre's source and duration of consumption in the rearing (González‐Alvarado et al. 2010). RH (Treatment 2) inclusion improved the FCR and BWG of broilers from 11 to 24 days of age, and SBP inclusion in the diet had no significant effect on FCR and BWG from 25 to 42 days of age in comparison to the control group. The results of the current experiment showed that T4 (1.5:1.5 ratio of RH to SBP) increased FI of broilers from 11 to 24 days of age compared to the T1 and T2 groups. It suggests that chicks fed the T4 diet had the lowest digesta viscosity compared to chicks fed the T1 and T2 diets (Figure 3). As a result, reducing the viscosity (T4) increases the passage rate and FI. Moreover, the T4 group had the highest BWG compared to other groups from 25 to 42 days of age. Higher levels of dietary insoluble fibre will accelerate the passage of digesta through the distal section of the gastrointestinal tract (GIT), perhaps leading to increased FI (Hetland et al. 2004). According to our findings, Saki et al. (2011) observed that supplementation with soluble and insoluble fibre (1.5% modified pectin and 1.5% cellulose) in the diet of broiler chickens enhanced daily FI from 1 to 21 days of age. On the other hand, Sadeghi et al. (2015) observed that the inclusion of 1.5% RH and 1.5% SBP in the broiler diet reduced daily FI and BWG from 14 to 28 days of age. It was demonstrated that including insoluble fibre such as wood shavings, soya bean hulls and oat hulls in broiler diets improved body weight and FCR (Amerah et al. 2009). In the current study, the FCR of broilers improved in the T2, T3 and T4 groups from 11 to 42 days of age compared to the control groups. Contrary to our results, supplementation with soluble fibre such as SBP reduced feed efficiency when provided in diets at 3% from 1 to 42 days of age (Sadeghi et al. 2015).

Some blood metabolites can be used to measure the health of broilers. GLU concentration changes in plasma represent a dynamic balance of absorption and metabolism (Geng et al. 2021). In the present experiment, supplementation of RH in the T2 group decreased the GLU concentration at 24 days of age. AbouSekken et al. (2013) observed that increasing dietary SBP levels from 1% to 5% significantly decreased GLU concentration in broiler chickens. In humans, eating water‐binding fibres decreases GLU absorption by changing the movement trend of the small intestine (Cherbut et al. 1994). The concentration of T‐CHO is a critical indicator for measuring lipid metabolism. In the present experiment, supplementation of RH in the T3 group decreased T‐CHO concentration in comparison with other groups at 42 days of age. It has been demonstrated that a portion of the insoluble fibre in rice bran acts as a lower agent of T‐CHO and coronary artery disease, and these effects are attributed to the tocotrienols in raw rice bran (Shirzadegan and Taheri 2017). In agreement with our findings, in broiler breeder hens, Mohiti‐Asli et al. (2012) reported that, in hens fed with a diet containing 3% cellulose, T‐CHO concentration was significantly reduced. AbouSekken et al. (2013) observed that the inclusion of 1%–5% SBP in a broiler diet had no significant effect on T‐CHO concentration. Supplementation of 0.75% insoluble fibre in a broiler diet from 1 to 42 days of age reduced TG and T‐CHO concentrations (Sarikhan et al. 2009). This effect can be attributed to a decrease in bile acid concentrations as a result of the binding effect of non‐starch polysaccharides and their ability to enhance bile acid excretion (Ohtani 2002).

Maintaining a correct pH in the GIT is critical for digestive enzyme activity. The inclusion of SBP (Treatment 3) in the diet reduced the pH of the gizzard and ilium of broilers at 24 days of age compared to the control group. This result may be due to the increasing SBP geometric diameter mean (GMD) in comparison to RH from 11 to 24 days of age. Coarse particles of DF sources remain in the gizzard for a long time, thereby increasing the muscular activity of this organ (Hetland and Svihus 2001), and causing the reduction observed in pH. In agreement with our findings, Jiménez‐Moreno et al. (2010) observed that chicks given SBP had more HCl in their gizzard digesta. On the other hand, Dos santos et al. (2019) observed that the inclusion of 2.5%–5% of RH and soya bean hulls in broiler chickens reduced the gizzard pH. A decrease in excreta moisture results in litter quality improvement. In Treatment 3, litter moisture decreased at 24 and 42 days of age compared to the control groups. The gizzard of chicks fed a fibre‐based diet would act as a pacemaker function for nutrient digestion and controlling water absorption to an ideal level, resulting in reduced water excretion (Naeem et al. 2023). Furthermore, the GIT microflora ferments soluble fibre, which improves the GIT environment and lowers the incidence of diarrhoea (Montagne et al. 2003).

Digestibility is a key indicator of the digestion of feed and animal performance. According to a study, adding oat hulls to the broiler chickens’ diet as a source of insoluble fibre could enhance the digestibility of EE, OM, DM and CP compared to the control diet without oat hulls (Kheravii, Swick, et al. 2018). The results in our present study showed that the digestibility of DM, OM and NDF increased in broiler chickens fed the T4 group in comparison to the T3 group at 42 days of age. It has been suggested that RH, as an insoluble fibre, can increase the size and weight of digestive organs (Tejeda and Kim 2020), whereas the effect of SBP, as a soluble fibre, which is more fermentable, increases the effectiveness of RH and improves nutrient digestibility in birds fed with the T4 group. Birds lack the enzymes required to break down dietary cellulose, hemicellulose and pectin. Therefore, these compounds are fermented by bacteria in the distal part of the digestive tract (Kheravii, Morgan et al. 2018). The T4 group probably had separate microbial compositions that facilitated fibre breakdown, resulting in a better digestibility of NDF than the other groups. At 42 days of age, Treatment 3 may have reduced DM and OM digestibility of birds fed with 3% RH due to increasing silica content in RH, which reduced enzyme activity (Jiménez‐Moreno et al. 2019).

The primary location of nutrient absorption is the intestinal villus, and the effectiveness of nutrient absorption and utilization is determined by the growth of the villus structure (Sklan et al. 2003). In the current trial, dietary supplementation of RH in the T2 group increased the VH at 24 days of age compared to the other groups. According to Zhang et al. (2023), VH can improve intestinal absorption capacity by increasing the surface area of epithelial cells in the small intestine. Contrary to our findings, Torki et al. (2018) found that the inclusion of 5% oat hull in broiler diet had no significant effect on VH in the ileum at 21 days of age. In the present experiment, dietary supplementation with SBP in the T3 group decreased the VH of theilium, whereas the T1 and T2 groups increased the VSA compared to the other groups at 42 days of age. Saki et al. (2011) observed that the addition of 2% pectin and 1% cellulose to the diet of broiler chickens reduced the VH and VSA of the jejunum at 14 days of age. Sadeghi et al. (2015) observed that feeding 3% RH increased VH in ileum at 21 days of age. Therefore, in Group 2, VSA increased both at 24 and 42 days of age. It appears that intestinal morphology improves with the inclusion of RH during the grower period and SBP during the finisher period. The secretion of digestive juices is the primary purpose of intestinal crypts, and CD represents the rate of cell proliferation. The crypt of cells became shallow, showing an increase in cell maturation rate and secretory activity (Chiou et al. 1996). In the current study, the CD of the ileum in the T1 group was significantly lower than that of the other groups at 24 days of age. The short‐chain fatty acids produced by fibre fermentation may promote intestinal cell proliferation and increase nutrient absorption (Namkung et al. 2011). In the current trial, dietary supplementation of SBP in the T3 group from 11 to 24 days of age and the T2 group at 25–42 days of age increased the ilium length. Previous research has found that the inclusion of soluble fibre is more connected with increasing intestine length than insoluble fibre. As an example, Jimenez‐Moreno et al. (2013) found that broiler chicks fed with SBP had longer intestines than those given RH. In the present experiment, the T1 group had a higher ilium viscosity and less VSA than other groups at 24 days of age. Higher viscosity may hinder nutrient diffusion, leading to decreased digestion and transfer by endogenous enzymes at the surface of the mucus (Bederska‐Łojewska et al. 2017).

## 5 Conclusion

According to the results of the present study, growth performance and ilium morphology were significantly improved in the T2 group at 24 days of age. In addition, in the T4 group viscosity of ilium contents reduced at 24 days of age, as well as BWG and nutrient digestibility at 42 days of age. On Day 24, the T3 group, which was fed with SBP, the pH of the ilium and litter moisture significantly decreased at 42 days of age. Furthermore, GLU and T‐CHO concentrations in the T2 and T3 groups decreased at 24 and 42 days of age, respectively. In the T3 group at 24 days of age and in the T2 group at 42 days of age, the ilium length was significantly reduced compared to other groups. In conclusion, the inclusion of RH in broiler diet from 11 to 24 days of age and SBP from 25 to 42 days of age (T2 group) and supplementation of 1.5% RH and 1.5% SBP from 11 to 42 days of age (T4 group) could improve broiler performance.

## Author Contributions

**Ali Asghar Kardel**: methodology, investigation, resources, writing – original draft. **Mohammad Kazemifard**: conceptualization, methodology, formal analysis, project administration, resources, writing – review and editing. **Mansour Rezaei**: data curation, formal analysis, writing – review and editing. **Asadollah Teimouri Yansari**: conceptualization, visualization, resources.

## Ethics Statement

All procedures were approved by the Animal Care and Use Committee of the Sari University of Agricultural Sciences and Natural Resources, Sari, Iran.

## Conflicts of Interest

The authors declare no conflicts of interest.

## Peer Review

The peer review history for this article is available at https://www.webofscience.com/api/gateway/wos/peer‐review/10.1002/vms3.70471.

## Data Availability

Upon reasonable request, the corresponding author will provide the data supporting the study's conclusions.
